# Improving calculation, interpretation and communication of familial colorectal cancer risk: Protocol for a randomized controlled trial

**DOI:** 10.1186/1748-5908-5-6

**Published:** 2010-01-28

**Authors:** Nicky Dekker, Rosella PMG Hermens, Glyn Elwyn, Trudy van der Weijden, Fokko M Nagengast, Peter van Duijvendijk, Simone Salemink, Eddy Adang, J Han JM van Krieken, Marjolijn JL Ligtenberg, Nicoline Hoogerbrugge

**Affiliations:** 1 Department of Human Genetics, Radboud University Nijmegen Medical Centre, Nijmegen, the Netherlands; 2 Scientific Institute for Quality of Healthcare, Radboud University Nijmegen Medical Centre, Nijmegen, the Netherlands; 3 Department of Primary Care and Public Health, Cardiff University, Cardiff, UK; 4 Department of General Practice, CAPHRI School for Public Health and Primary Care, Maastricht University, Maastricht, the Netherlands; 5 Department of Gastroenterology, Radboud University Nijmegen Medical Centre, Nijmegen, the Netherlands; 6 Department of Surgery, Radboud University Nijmegen Medical Centre, Nijmegen, the Netherlands; 7 Department of Epidemiology, Biostatistics and HTA assessment, Radboud University Nijmegen Medical Centre, Nijmegen, the Netherlands; 8 Department of Pathology, Radboud University Nijmegen Medical Centre, Nijmegen, the Netherlands; 9 Department of Medical Oncology, Radboud University Nijmegen Medical Centre, Nijmegen, the Netherlands

## Abstract

**Background:**

Individuals with multiple relatives with colorectal cancer (CRC) and/or a relative with early-onset CRC have an increased risk of developing CRC. They are eligible for preventive measures, such as surveillance by regular colonoscopy and/or genetic counselling. Currently, most at-risk individuals do not follow the indicated follow-up policy. In a new guideline on familial and hereditary CRC, clinicians have new tasks in calculating, interpreting, and communicating familial CRC risk. This will lead to better recognition of individuals at an increased familial CRC risk, enabling them to take effective preventive measures. This trial compares two implementation strategies (a common versus an intensive implementation strategy), focussing on clinicians' risk calculation, interpretation, and communication, as well as patients' uptake of the indicated follow-up policy.

**Methods:**

A clustered randomized controlled trial including an effect, process, and cost evaluation will be conducted in eighteen hospitals. Nine hospitals in the control group will receive the common implementation strategy (*i.e*., dissemination of the guideline). In the intervention group, an intensive implementation strategy will be introduced. Clinicians will receive education and tools for risk calculation, interpretation, and communication. Patients will also receive these tools, in addition to patient decision aids. The effect evaluation includes assessment of the number of patients for whom risk calculation, interpretation, and communication is performed correctly, and the number of patients following the indicated follow-up policy. The actual exposure to the implementation strategies and users' experiences will be assessed in the process evaluation. In a cost evaluation, the costs of the implementation strategies will be determined.

**Discussion:**

The results of this study will help determine the most effective method as well as the costs of improving the recognition of individuals at an increased familial CRC risk. It will provide insight into the experiences of both patients and clinicians with these strategies.

The knowledge gathered in this study can be used to improve the recognition of familial and hereditary CRC at both the national and international level, and will serve as an example to improve care for patients and their relatives worldwide. Our results may also be useful in improving healthcare in other diseases.

**Trial registration:**

ClinicalTrials.gov NCT00929097

## Background

The lifetime risk of developing colorectal cancer (CRC) in Western society is 5 to 6%[1,2]. Familial and hereditary cancers account for approximately 15 to 20% of all CRCs [3-5]. In these families, healthy relatives of CRC patients may have an increased risk of developing CRC themselves. This so-called familial CRC risk can be divided into three groups, based on cumulative lifetime risks of developing CRC:

1. Average: familial CRC risk below 10%.

2. Moderate: familial CRC risk of 10-15%.

3. High: familial CRC risk above 15%.

For each group, a different follow-up policy applies. For individuals with an average familial CRC risk, neither surveillance nor genetic counselling is indicated. For individuals with a moderate familial CRC risk, surveillance by regular colonoscopy is indicated. For individuals with a high familial CRC risk, referral for genetic counselling is recommended. Identification of individuals with an increased familial CRC risk is crucial because surveillance significantly reduces CRC-related morbidity and mortality, by 43 to 80% and 65 to 81%, respectively[6,7]. Both underuse and overuse of surveillance and genetic counselling have a significant impact on patients and their relatives, and may lead to unnecessary costs.

Familial CRC risk is assessed by family history and, in a subset of patients, microsatellite instability (MSI) analysis performed by pathologists. Unfortunately, both procedures are difficult. Previous research has shown that patient family history often is missing or incomplete, and information provided by patients is not always accurate[4,8-11]. Furthermore, interpretation of the family history (determining the indicated follow-up policy) is not always correct[12]. Pathologists' selection of patients for MSI is often incomplete, while clinicians regularly interpret the results incorrectly [Overbeek LI, Hermens RP, van Krieken JH, Adang E, Casparie M, Akkermans R, Nagengast FM, Ligtenberg MJ, Hoogerbrugge N. A tailored implementation strategy increases involvement of pathologists in the recognition of patients at risk for Lynch syndrome: cluster randomised controlled trial, submitted] Consequently, only 12 to 30% of CRC patients with a high familial CRC risk are referred for genetic counselling[4,10,13-15]. Other studies have shown that many CRC patients referred to a familial cancer clinic belong to an average or moderate risk population for whom genetic counselling is not indicated[16,17].

Clinicians involved in the care for CRC patients recognize the need for improvement in this area. Therefore, a multidisciplinary evidence-based guideline on familial and hereditary colorectal cancer (FHCC) was launched in the Netherlands in 2008[18]. An important addition compared to previous national and international guidelines is that surgeons and gastroenterologists (referred to as 'clinicians' in this protocol) have new tasks in calculating, interpretating, and communicating familial CRC risk. Because clinicians are often unfamiliar with these tasks, implementation strategies are needed to ensure that patients and their relatives receive proper counselling and follow-up[14]. In a previous trial, an electronic reminder system specifically aimed at pathologists improved completeness of patient selection for MSI testing [Overbeek LI, Hermens RP, van Krieken JH, Adang E, Casparie M, Akkermans R, Nagengast FM, Ligtenberg MJ, Hoogerbrugge N. A tailored implementation strategy increases involvement of pathologists in the recognition of patients at risk for Lynch syndrome: cluster randomised controlled trial, submitted] In this trial, we will provide support at both clinician and patient level to further implement the guideline.

This trial compares two implementation strategies: a common strategy (*i.e*., dissemination of the guideline) versus an intensive implementation strategy, focussing on clinicians' risk calculation, interpretation, and communication, as well as patients' uptake of the indicated follow-up policy.

An effect, process, and cost evaluation will be performed. The improvement of the identification and referral of patients at an increased familial CRC risk will lead to a higher number of individuals following an appropriate surveillance program, thereby reducing CRC-related morbidity and mortality.

## Methods

### Study design and setting

A clustered randomized controlled trial including an effect, process, and cost evaluation will be conducted in eighteen community hospitals. All patients with CRC diagnosed under the age of 70 years and their clinicians will be invited to participate. To prevent contamination bias, randomization will take place at hospital level. Stratification will take place according to hospital size (<500, 500 to 700, and >700 beds), and be performed by means of a computerized randomization system. This study was approved by the Committee on Research Involving Human Subjects of the region Arnhem-Nijmegen.

### Primary objectives

This trial compares two implementation strategies: a common strategy versus an intensive implementation strategy, focussing on clinicians' risk calculation, interpretation, and communication, as well as patients' uptake of the indicated follow-up policy.

### Hypothesis

Providing patients and clinicians with information on CRC, a risk assessment tool, risk communication aids, and decision aids will improve calculation, interpretation, and communication of the familial CRC risk by clinicians, as well as patients' uptake of the indicated follow-up policy more than dissemination of the guideline only.

### Outcome measures

#### Effect evaluation

1. The percentage of CRC patients for whom a correct familial CRC risk is calculated by clinicians.

2. The percentage of CRC patients for whom a calculated familial CRC risk is correctly interpreted by clinicians.

3. The percentage of CRC patients with whom a calculated familial CRC risk and/or follow-up policy is communicated by clinicians.

4. Patients' uptake of the indicated follow-up policy.

#### Process evaluation

1. Actual exposure to the different elements of the implementation strategies.

2. The experiences of patients and clinicians with these elements.

#### Cost evaluation

Costs of the implementation strategies in relation to the number of correctly referred patients.

#### Participants

Clinicians from eighteen hospitals will participate. All their patients diagnosed with CRC under the age of 70 years during the six-month inclusion period are eligible for inclusion. Patients must be able to provide informed consent and be able to read and understand Dutch. Patients previously referred for genetic counselling for CRC are excluded. Patients will be selected by PALGA (Pathologisch-Anatomisch Landelijk Geautomatiseerd Archief), the nationwide network and registry of histo- and cytopathology in the Netherlands[19].

All patients will receive a patient information letter, signed by their treating clinician, along with an informed consent form. After signing the informed consent form, they will be included in the study.

### Interventions

#### Implementation strategies in both groups

In both the control group and the intervention group, clinicians will receive the FHCC guideline.

#### Intensive implementation strategy in the intervention group

**Figure 1:**
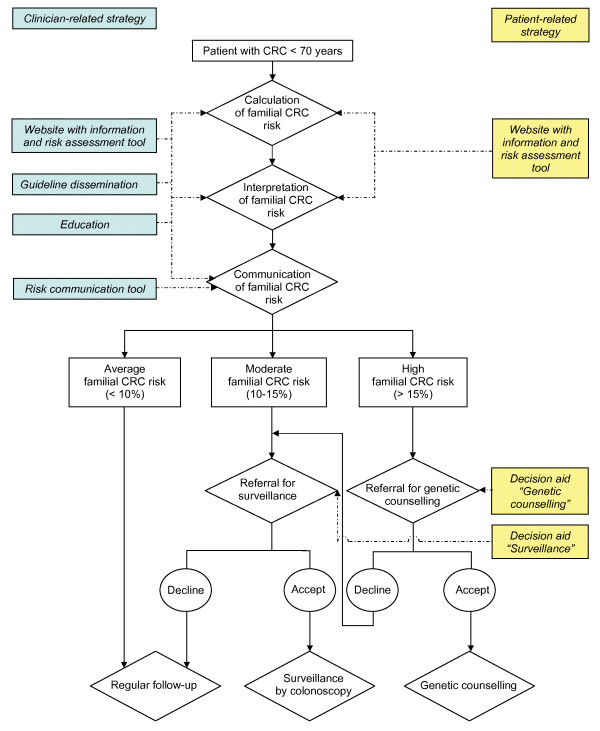
**Intensive implementation strategy for the intervention group, aimed at both patients and clinicians**. The rhombuses in this figure represent the tasks clinicians have in calculation, interpretation and communication of the familial colorectal cancer (CRC) risk in CRC patients. It also shows the various elements of the intensive implementation strategy aimed at both patients and clinicians (in yellow and green, respectively) that will be compared to dissemination of the guideline on familial and hereditary colorectal cancer only. Familial CRC risk: cumulative lifetime risk of developing CRC for first-degree relatives of CRC patients.

The intensive implementation strategy is summarized in Figure 1 and consists of the following implementation tools: a website for patients and clinicians; education for clinicians; and a risk communication tool for clinicians.

#### Website

The website consists of the following items:

1. A summary of evidence-based and relevant information about familial CRC risk (lifetime risk of developing CRC). Natural frequencies with the same denominator and visual displays will be used. Absolute risks will be presented, as well as in comparison to the population risk. The outcomes are offered in both positive and negative frames (*e.g*., the risk of developing CRC as well as the chance of not developing CRC). The focus is on familial CRC risk and the different follow-up policies. The information is presented in two different formats, one for patients and one for clinicians.

2. A risk assessment tool to calculate patients' familial CRC risk. Patients fill in medical information about themselves and their relatives with regard to CRC and other cancers. Clinicians can use the tool as well. The calculated familial CRC risk is given in the same format as the rest of the website. Advice for follow-up is offered based on this risk, as well as a reminder to use the corresponding decision aid, if applicable.

3. Decision aids, aimed at facilitating decisions involving the uptake of the indicated follow-up policy (one for surveillance and one for genetic counselling). Tools supporting patients in making informed choices about their healthcare, such as decision aids, have been shown to improve knowledge, clarify preferences, and reduce uncertainty around decision making, with high levels of acceptability among consumers[20,21]. The decision aids used in this trial provide balanced information on different options, *i.e*., to be referred for surveillance/genetic counselling or not. The following items are addressed: background information, benefits and harms, and the potential impact on the patient and their relatives. Worksheets are provided for patients to list and rate the importance of the benefits and harms for themselves.

The website is available exclusively to patients and clinicians in the intervention group. A login name and password will be provided upon inclusion. Patients can use the website independently before or after regular follow-up visits, and are encouraged to discuss the results with their clinician. They are instructed to keep the decision aid within their family, and not share it for reasons of research integrity. To minimize contamination bias, after the trial all patients in the control group will be asked if they were exposed to the website.

#### Education

In an educational meeting, clinicians in the intervention group will be trained to use the FHCC guideline.

#### Risk communication tool

Clinicians will receive a tool for communicating the familial CRC risk with their patients during a regular follow-up visit. The tool consists of written information and visual displays of the population risk of CRC, an explanation of the risk level of the patient and his/her relatives, as well as the indicated follow-up policy. It is designed in the same format as the website.

#### Development of the implementation tools

During development, the content and presentation of the website and the risk communication tool will be reviewed by physicians not specifically trained in genetics, and by non-medical personnel as well as representatives from the Dutch CRC patient associations (Vereniging HNPCC-Lynch and Stichting Doorgang). Improvements will be made based on their comments. Before use, the tools will be tested among approximately 20 patients and 20 clinicians. The purpose of this pilot is determining whether the website and the tools are acceptable, the information is presented clearly, and the completion of the tools is feasible.

#### Power calculation

To detect a difference of 20% between the intervention group and the control group in uptake of surveillance by colonoscopy in patients at moderate familial CRC risk, and referral for genetic counselling in patients at a high familial CRC risk, at least 186 patients are required (alpha = 0.05, a two-sided testing and power = 0.80). However, considering an intracluster-correlation coefficient of 0.15 and an average of five patients per clinician, at least sixty clinicians and 300 patients are needed. For eighteen hospitals this means three to four clinicians and 15 to 20 patients per hospital.

### Data collection

#### Baseline characteristics

Baseline characteristics from patients, clinicians, and hospitals will be collected in the following manner:

1. Patients: From PALGA, data including age, gender, and some medical information will be collected. Medical information includes diagnoses of cancer (diagnosed since 1971), cancer type, and age at diagnosis, as well as the result of MSI testing. The following data will be collected by a self-administered questionnaire: ethnicity, current marital status, educational level, previous medical or health training, and family history of cancer (type of cancer and age at diagnosis). Family history is collected for first-degree relatives (*i.e*., parents, siblings, and children).

2. Clinicians: All participating clinicians will be asked to provide baseline data (*e.g*., specialization, number of years of experience) in a questionnaire

3. Hospitals: From the hospitals' websites, characteristics such as size, teaching status, and presence of an outpatient department for genetic counselling will be obtained.

#### Effect evaluation

Before introducing the implementation strategies, a baseline assessment of risk calculation, interpretation, and communication will be performed. Both the baseline assessment and the evaluation of the implementation strategies will be performed retrospectively in the same manner. Baseline characteristics will be collected in the manner described above. The measuring instruments will be developed by identifying all relevant variables and translating these into questionnaires. When possible, existing validated questionnaires will be used.

The family history as reported by the patient in the questionnaire will be used to calculate a formal familial CRC risk and determine the indicated follow-up policy. This will be compared to the family history taken by the clinician, along with the risk calculation and interpretation performed by the clinician. These data will be extracted from the patients' medical records. The medical records will also be used to determine the number of patients with whom the familial CRC risk and corresponding follow-up policy has been communicated. To determine the number of referred patients who actually visit a familial cancer clinic, these clinics will be asked to report whether these patients have visited. The uptake of surveillance by colonoscopy by first-degree relatives will be determined by asking the patients whether their relatives are actually screened. Medical records and results from the decision aids on the website will be used to determine whether patients at an increased risk who were not referred for surveillance or genetic counselling were not referred because they had chosen not to be referred or because it was not discussed.

#### Process evaluation

In the process evaluation, data are collected on actual exposure of patients and clinicians to the different elements of the implementation strategies, as well as their experience with these elements:

1. Website: The website automatically records the following data when it is used: who used which elements; how often did users visit the different elements of the website; and how long did it take to use the different elements. By using questionnaires, users' experiences with the website will be ascertained.

2. Education: Attendance at the meetings will be determined by keeping an attendance list. The duration of the meetings will be recorded. In addition, clinicians' experience with the meetings will be ascertained by using a questionnaire.

3. Risk communication tool: Patients and clinicians will be asked whether the tool was used. Their experience with the tool will be measured using questionnaires focussing on the perceived usefulness and usability of the tool.

#### Cost evaluation

Costs accompanied with the development and implementation of the website and risk communication tool will be accounted for, as well as the costs for dissemination of the guideline. Clinicians will provide time estimates to use the different elements. Costs will be correlated to the number of correctly referred patients.

### Data analysis

#### Effect evaluation

To analyze the effectiveness of both implementation strategies, descriptive statistics and multilevel analysis will be used. Patient, clinician, and hospital characteristics will be included in the multilevel analysis, allowing for correction of the effectiveness for probable differences in case mix between the different hospitals. The statistical analyses will be performed using SPSS version 16.0 for Windows.

The percentage of correctly referred patients is defined as follows:

1. The percentage of patients at an average familial CRC risk who are not referred for surveillance or genetic counselling.

2. The percentage of patients at a moderate familial CRC risk who want to be referred and are referred for surveillance.

3. The percentage of patients at a moderate familial CRC risk who do not want to be referred and are not referred for surveillance.

4. The percentage of patients at a high familial CRC risk who want to be referred and are referred for genetic counselling.

5. The percentage of patients at a high familial CRC risk who do not want to be referred and are not referred for genetic counselling but are referred for surveillance if they opt for it.

6. The percentage of patients at a high familial CRC risk who do not want to be referred and are not referred for genetic counselling or surveillance.

#### Process evaluation

Frequencies and means are used to assess the actual exposure of the patients and clinicians to the elements of the implementation strategies and to assess their experience with these elements. A multilevel regression analysis will be applied to assess which elements of the intensive implementation strategy were particularly associated with effective implementation of the new FHCC guideline.

#### Cost evaluation

The costs of implementation related resource use will be calculated on a per patient basis. The costs of the use of each element per correctly referred patient will be calculated. The costs of the intensive implementation strategy will be compared to the costs of dissemination of the guideline only.

## Discussion

### Objectives

The aim of this trial is to compare two implementation strategies: a common implementation strategy (dissemination of the guideline only) versus an intensive implementation strategy, focussing on clinicians' risk calculation, interpretation, and communication, as well as patients' uptake of the indicated follow-up policy, such as referral for colonoscopy or genetic counselling.

### Strengths and weaknesses

To our knowledge, this is the first study of an implementation strategy designed to improve the recognition of patients' familial CRC risk by addressing both patients and their clinicians (surgeons and gastroenterologists). If the intensive implementation strategy is successful, the elements (the website and the risk communication tool) can be released for general use by patients and clinicians. They may also serve as an example for other hereditary and non-hereditary diseases. Our study will add knowledge to the effectiveness of patient decision aids and the best way of supplying patients and clinicians with information on disease risks.

Some limitations need to be addressed. Family history as reported by the clinician will be compared to the family history reported by the patient in a self-administered questionnaire. Previous research has shown that the accuracy of family history of CRC in first-degree relatives reported by patients is approximately 90%[22]. The optimal way of ensuring that the family history reported by the patient is accurate is by reviewing medical records of the affected relatives. Since written permission from relatives is needed to do so, this is not feasible and will therefore not be done in this study.

In our evaluation, only patients will be included; their relatives are not. Patients will be asked whether their relatives are screened, but the relatives will not be contacted to assess whether they actually received the results from the risk assessment and the matching advice.

Measurements may be contaminated in case others are provided with the login code for the website.

Collecting data from medical records does not monitor everything that is discussed between the clinician and the patient. This may lead to underestimation of the risk interpretation and communication. Videotaping the consultations would shed light on the content and quality of the risk communication, but would also influence the intervention by reminding the clinician of the intervention. In this study, regular clinical practice will be left undisturbed as much as possible.

### Implications

The results of this study will help determine the most effective way of improving the recognition of individuals at an increased familial CRC risk. It will provide insight into the experiences of both patients and clinicians with these strategies.

This is important because many people are currently not treated according to evidence-based guidelines, and can benefit from proper cancer risk assessment and appropriate follow-up, which has been proven to reduce morbidity and mortality. The knowledge gathered in this study may improve the recognition of familial and hereditary CRC at national and international level and serve as an example to improve care for patients and their relatives worldwide. In addition, our results may be useful in improving healthcare in other diseases.

## Competing interests

The authors declare that they have no competing interests.

## Authors' contributions

ND drafted the protocol, created the intervention materials, and is involved in the implementation, analysis, and reporting aspects of the study. NH and RH, the project leaders, conceived, designed, and obtained funding for the study and are involved in all aspects of the study. GE and TvdW advised the project team on shared decision making. HvK, FN, PvD, EA and ML participated in designing the study. SS provided content expertise for the intervention materials. EA provided advice on the economic evaluation. All authors have read and approved the final manuscript.
